# Supplementary material for: P4-ATPases control phosphoinositide membrane asymmetry and neomycin resistance
Source: Nat Cell Biol. 2025 Jul 11;27(7):1114–24. doi: 10.1038/s41556-025-01692-z (PMC12270916; doi:10.1038/s41556-025-01692-z)
Supplement: Supplementary file 1 — Supplementary Tables 1 and 2. [file 41556_2025_1692_MOESM1_ESM.pdf]

nature cell biology

Supplementary information

https://doi.org/10.1038/s41556-025-01692-z

# P4-ATPases control phosphoinositide membrane asymmetry and neomycin resistance

In the format provided by the authors and unedited

**Supplementary Table 1. Cryo-EM data collection, refinement and validation statistics**

| | Neo1 in E2P state bound with PI4P (EMD-44850) (PDB 9BS1) |
|---|---|
| **Data collection and processing** | |
| Magnification | 105,000 |
| Voltage (keV) | 300 |
| Electron dose ($e^-$/Å$^2$) | 58 |
| Defocus range (-μm) | 1.3-1.6 |
| Pixel size (Å) | 0.828 |
| Symmetry imposed | C1 |
| Initial particle images (no.) | 6,050,356 |
| Final particle images (no.) | 1,082,029 |
| Map resolution (Å) | 3.7 |
| FSC threshold | 0.143 |
| Map resolution range (Å) | 1.8-10.6 |
| **Refinement** | |
| Initial model used (PDB code) | AF-P40527-F1-model_v4 |
| Model resolution (Å) | 4.0 |
| FSC threshold | 0.5 |
| Map sharpening *B* factor (Å$^2$) | -175.3 |
| Model composition | |
| Non-hydrogen atoms | 7,760 |
| Protein residues | 965 |
| Ligands | 2 |
| *B* factors (Å$^2$) | |
| Protein | 69.39 |
| Ligand | 20.00 |
| R.m.s. deviations | |
| Bond lengths (Å) | 0.003 |
| Bond angles (°) | 0.621 |
| **Validation** | |
| MolProbity score | 1.98 |
| Clashscore | 9.51 |
| Poor rotamers (%) | 0.23 |
| Ramachandran plot | |
| Favored (%) | 92.20 |
| Allowed (%) | 7.80 |
| Disallowed (%) | 0.00 |

**Supplementary Table 2: Yeast strains and plasmids used in the study.**

| Strain | Genotype | Plasmid | Source |
|---|---|---|---|
| BKJ424 | MATa *his3Δ1 leu2Δ0* ura3Δ0 lys2Δ0*neo1Δ*::KanMX | pRS313-NEO1 | Bai, L. et al., 2021 |
| BKJ426 | MATa *his3Δ1 leu2Δ0* ura3Δ0 lys2Δ0*neo1Δ*::KanMX | pRS313-NEO1 Q209G | Bai, L. et al., 2021 |
| BKJ427 | MATa *his3Δ1 leu2Δ0* ura3Δ0 lys2Δ0*neo1Δ*::KanMX | pRS313-Neo1 S221A | Bai, L. et al., 2021 |
| BKJ428 | MATa *his3Δ1 leu2Δ0* ura3Δ0 lys2Δ0*neo1Δ*::KanMX | pRS313-Neo1 S221L | Bai, L. et al., 2021 |
| BKJ429 | MATa *his3Δ1 leu2Δ0* ura3Δ0 lys2Δ0*neo1Δ*::KanMX | pRS313-Neo1 S452A | Bai, L. et al., 2021 |
| BKJ430 | MATa *his3Δ1 leu2Δ0* ura3Δ0 lys2Δ0*neo1Δ*::KanMX | pRS313-Neo1 S452Q | Bai, L. et al., 2021 |
| BKJ432 | MATa *his3Δ1 leu2Δ0* ura3Δ0 lys2Δ0*neo1Δ*::KanMX | pRS313-Neo1 T453S | Bai, L. et al., 2021 |
| BKJ433 | MATa *his3Δ1 leu2Δ0* ura3Δ0 lys2Δ0*neo1Δ*::KanMX | pRS313-Neo1 K236A | Bai, L. et al., 2021 |
| BKJ434 | MATa *his3Δ1 leu2Δ0* ura3Δ0 lys2Δ0*neo1Δ*::KanMX | pRS313-Neo1 K236R | Bai, L. et al., 2021 |
| BKJ435 | MATa *his3Δ1 leu2Δ0* ura3Δ0 lys2Δ0*neo1Δ*::KanMX | pRS313-Neo1 E237A | Bai, L. et al., 2021 |
| BKJ436 | MATa *his3Δ1 leu2Δ0* ura3Δ0 lys2Δ0*neo1Δ*::KanMX | pRS313-Neo1 E237D | Bai, L. et al., 2021 |
| BKJ437 | MATa *his3Δ1 leu2Δ0* ura3Δ0 lys2Δ0*neo1Δ*::KanMX | pRS313-Neo1 R247A | Bai, L. et al., 2021 |
| BKJ438 | MATa *his3Δ1 leu2Δ0* ura3Δ0 lys2Δ0*neo1Δ*::KanMX | pRS313-Neo1 R247L | Bai, L. et al., 2021 |
| BKJ439 | MATa *his3Δ1 leu2Δ0* ura3Δ0 lys2Δ0*neo1Δ*::KanMX | pRS313-Neo1 S488A | Bai, L. et al., 2021 |
| BKJ440 | MATa *his3Δ1 leu2Δ0* ura3Δ0 lys2Δ0*neo1Δ*::KanMX | pRS313-Neo1 S488W | Bai, L. et al., 2021 |
| BKJ441 | MATa *his3Δ1 leu2Δ0* ura3Δ0 lys2Δ0*neo1Δ*::KanMX | pRS313-Neo1 Q193A | Bai, L. et al., 2021 |
| BKJ443 | MATa *his3Δ1 leu2Δ0* ura3Δ0 lys2Δ0*neo1Δ*::KanMX | pRS313-Neo1 P456A | Bai, L. et al., 2021 |
| BKJ481 | MATa *his3Δ1 leu2Δ0* ura3Δ0 lys2Δ0*neo1Δ*::KanMX | pRS313-Neo1 I484A | This study |
| BKJ482 | MATa *his3Δ1 leu2Δ0* ura3Δ0 lys2Δ0*neo1Δ*::KanMX | pRS313-Neo1 V485A | This study |
| BY4741 | *MATa his3 leu2 ura3 met15* | | Invitrogen |

| | | | |
|---|---|---|---|
| ZHY615M2D | *MATα his3 leu2 ura3 lys2 drs2Δ* | | Hua, Z. et al., 2002 |
| PFY3275F | MATa his3 leu2 ura3 met15 *dnf1Δ dnf2Δ* | | Hua, Z. et al., 2002 |
| BY4741 YMR162C | *MATa his3 leu2 ura3 met15 dnf3Δ* | | Invitrogen |
| ZHY628-15B | MATα his3 leu2 ura3 *neo1Δ* p413-neo*1-1* | | Hua,Z. et al., 2003, Takar,M et al., 2016 |
| TSA854 | MATa dop1-1::kanMX his3Δ1 leu2Δ0 ura3Δ0 met15Δ0 CAN1+ LYP1+ | | van Leeuwen, J. *et al.*,2016 |
| Y14541 | MAT@ mon2Δ::kanMX his3Δ1 leu2Δ0 ura3Δ0 lys2Δ0 + [GALpr-MON2, URA3] | | van Leeuwen, J. *et al.*,2016 |
| AAY102 | SEY6210; *stt4Δ*::*HIS3* carrying pRS415*stt4-4* (*LEU2 CEN6 stt4-4*) | | Audhya, A. et al., 2000 |
| BKJ810 | SEY6210; *stt4Δ*::*HIS3 neo1Δ* pRS414-Neo1S221L carrying pRS415*stt4-4* (*LEU2 CEN6 stt4-4*) | pRS314-Neo1 S221L | This study |
| BKJ811 | SEY6210; *stt4Δ*::*HIS3 neo1Δ* pRS414-Neo1S452QLcarrying pRS415*stt4-4* (*LEU2 CEN6 stt4-4*) | pRS314-Neo1 S221L | This study |
| BKJ812 | SEY6210; *stt4Δ*::*HIS3 neo1Δ* pRS414-Neo1carrying pRS415*stt4-4* (*LEU2 CEN6 stt4-4*) | pRS314-Neo1 | This study |
| AAY104 | SEY6210; pik1Δ::HIS3 carrying pRS314pik1-83 (TRP1 CEN6 pik1-83) | | Audhya, A. et al., 2000 |
| BKJ813 | SEY6210; pik1Δ::HIS3 carrying pRS314pik1-83 (TRP1 CEN6 pik1-83) | pRS315-Neo1 | This study |
| BKJ814 | SEY6210; pik1Δ::HIS3 carrying pRS314pik1-83 (TRP1 CEN6 pik1-83) | pRS315-Neo1S221L | This study |
| BKJ815 | SEY6210; pik1Δ::HIS3 carrying pRS314pik1-83 (TRP1 CEN6 pik1-83) | pRS315-Neo1S452Q | This study |
| AAY202 | SEY6210;_*mss4Δ::HIS3MX6* YC*plac*111*mss4*$^{ts}$*-102* (*LEU2 CEN6 mss4*$^{ts}$*-102*) | | Audhya, A. et al., 2002 |
| BKJ816 | SEY6210;_*mss4Δ::HIS3MX6* YC*plac*111*mss4*$^{ts}$*-102* (*LEU2 CEN6 mss4*$^{ts}$*-102*) | pRS314-Neo1 | This study |
| BKJ817 | SEY6210;_*mss4Δ::HIS3MX6* YC*plac*111*mss4*$^{ts}$*-102* (*LEU2 CEN6 mss4*$^{ts}$*-102*) | pRS314-Neo1S221L | This study |
| BKJ818 | SEY6210;_*mss4Δ::HIS3MX6* YC*plac*111*mss4*$^{ts}$*-102* (*LEU2 CEN6 mss4*$^{ts}$*-102*) | pRS314-Neo1S452Q | This study |

| | | | |
|---|---|---|---|
| BKJ819 | SEY6210; *stt4Δ*::*HIS3* carrying pRS415*stt4-4* (*LEU2 CEN6 stt4-4*) | pRS416-GFP-$\text{SidC}_{\text{P4C}}$ | This study |
| BKJ820 | SEY6210; pik1Δ::HIS3 carrying pRS314pik1-83 (TRP1 CEN6 pik1-83) | pRS416-GFP-$\text{SidC}_{\text{P4C}}$ | This study |
| BKJ821 | SEY6210; *mss4Δ::HIS3MX6* YC*plac*111$mss4^{ts}$*-102* (*LEU2 CEN6* $mss4^{ts}$*-102*) | pRS416-GFP-$\text{SidC}_{\text{P4C}}$ | This study |
| BKJ822 | *MATa his3 leu2 ura3 met15 sac1Δ*::NAT | | This study |
| BKJ823 | *MATa his3 leu2 ura3 met15 sac1Δ*::NAT | pRS423 | |
| BKJ824 | *MATa his3 leu2 ura3 met15 sac1Δ*::NAT | pRS423-Neo1 | This study |
| YWY10 | MATa *his3Δ1 leu2Δ0* ura3Δ0 lys2Δ0*neo1Δ*::KanMX | pRS416-Neo1 | This study |
| BKJ825 | MATa *his3Δ1 leu2Δ0* ura3Δ0 lys2Δ0*neo1Δ*::KanMX *sac1Δ*:HIS3MX6 | pRS416-Neo1 | This study |
| BKJ826 | MATa *his3Δ1 leu2Δ0* ura3Δ0 lys2Δ0*neo1Δ*::KanMX *sac1Δ*:HIS3MX6 | pRS416-Neo1 pRS315-Neo1 | This study |
| BKJ827 | MATa *his3Δ1 leu2Δ0* ura3Δ0 lys2Δ0*neo1Δ*::KanMX *sac1Δ*:HIS3MX6 | pRS416-Neo1 pRS315-*neo1-1* | This study |
| BKJ828 | MATa *his3Δ1 leu2Δ0* ura3Δ0 lys2Δ0*neo1Δ*::KanMX *sac1Δ*:HIS3MX6 | pRS416-Neo1 pRS315-*neo1-2* | This study |
| BY4741*osh1Δ* | *MATa his3 leu2 ura3 met15 osh1Δ* | | Invitrogen |
| BY4741*osh2Δ* | *MATa his3 leu2 ura3 met15 osh2Δ* | | Invitrogen |
| BY4741*osh3Δ* | *MATa his3 leu2 ura3 met15 osh3Δ* | | Invitrogen |
| BY4741*osh5Δ* | *MATa his3 leu2 ura3 met15 osh5Δ* | | Invitrogen |
| BY4741*osh6Δ* | *MATa his3 leu2 ura3 met15 osh6Δ* | | Invitrogen |
| BY4741*osh7Δ* | *MATa his3 leu2 ura3 met15 osh7Δ* | | Invitrogen |
| BY4741*osh4Δ* | *MATa his3 leu2 ura3 met15 osh4*Δ | | Invitrogen |
| BMY046a | ZHY628-15B (*neo1-1*) *osh4*Δ::LEU2 | | |
| BMY043a | ZHY907-5E (*neo1*Δ pNEO1::URA3) *osh4*Δ::LEU2 | | |
| BKJ829 | MATa *his3Δ1 leu2Δ0* ura3Δ0 lys2Δ0*neo1Δ*::KanMX *osh6Δ*:HIS3MX6 | pRS315-Neo1 | This study |
| BKJ830 | MATa *his3Δ1 leu2Δ0* ura3Δ0 lys2Δ0*neo1Δ*::KanMX *osh6Δ*:HIS3MX6 | pRS315-Neo1S221L | This study |
| BKJ831 | MATa *his3Δ1 leu2Δ0* ura3Δ0 lys2Δ0*neo1Δ*::KanMX *osh6Δ*:HIS3MX6 | pRS315-Neo1S452Q | This study |
| BKJ832 | MATa *his3Δ1 leu2Δ0* ura3Δ0 lys2Δ0*neo1Δ*::KanMX *osh6Δ*:HIS3MX6 | pRS315-*neo1-1* | This study |
| BKJ833 | MATa *his3Δ1 leu2Δ0* ura3Δ0 lys2Δ0*neo1Δ*::KanMX *osh1Δ*:HIS3MX6 | pRS315-*neo1-1* | This study |
| BKJ834 | MATa *his3Δ1 leu2Δ0* ura3Δ0 lys2Δ0*neo1Δ*::KanMX *osh2Δ*:HIS3MX6 | pRS315-*neo1-1* | This study |

| | | | |
|---|---|---|---|
| BKJ835 | MATa *his3Δ1 leu2Δ0* ura3Δ0 lys2Δ0*neo1Δ*::KanMX *osh3Δ*:HIS3MX6 | pRS315-*neo1-1* | This study |
| BKJ836 | MATa *his3Δ1 leu2Δ0* ura3Δ0 lys2Δ0*neo1Δ*::KanMX *osh5Δ*:HIS3MX6 | pRS315-*neo1-1* | This study |
| BKJ837 | MATa *his3Δ1 leu2Δ0* ura3Δ0 lys2Δ0*neo1Δ*::KanMX *osh7Δ*:HIS3MX6 | pRS315-*neo1-1* | This study |
| *sec12-4 neo1-1* | *MATα leu2 ura3 his3 trp1 lys2 sec12-4 neo1-1::HIS3-KanMX* | | Hua,Z. et al., 2003 |
| *sec14-1 neo1-1* | *MATa leu2 his3 lys2 sec14-1 neo1-1::HIS3-KanMX* | | Hua,Z. et al., 2003 |
| *sec18-1 neo1-1* | *MATα ura3 leu2 his3 trp1 lys2 suc2 sec18-1 neo1-1::HIS3-KanMX* | | Hua,Z. et al., 2003 |
| *sec21-1 neo1-1* | *MATa leu2 ura3 his3 trp1 suc2 sec21-1 neo1-1::HIS3-KanMX* | | Hua,Z. et al., 2003 |
| BKJ838 | MATa his3Δ1 leu2Δ0 ura3Δ0 lys2Δ0neo1Δ::KanMX | pRS313-Neo1 H472A | This study |
| BKJ839 | MATa his3Δ1 leu2Δ0 ura3Δ0 lys2Δ0neo1Δ::KanMX | pRS313-Neo1 H472I | This study |
| BKJ840 | MATa his3Δ1 leu2Δ0 ura3Δ0 lys2Δ0neo1Δ::KanMX | pRS313-Neo1 E475A | This study |
| BKJ841 | MATa his3Δ1 leu2Δ0 ura3Δ0 lys2Δ0neo1Δ::KanMX | pRS313-Neo1 H476A | This study |
| BKJ842 | MATa his3Δ1 leu2Δ0 ura3Δ0 lys2Δ0neo1Δ::KanMX | pRS313-Neo1 EH-AA | This study |
| BKJ843 | MATa his3Δ1 leu2Δ0 ura3Δ0 lys2Δ0neo1Δ::KanMX | pRS313-Neo1 EH-GG | This study |
| BKJ844 | *MATa his3Δ1 leu2Δ0 ura3Δ0 lys2Δ 0neo1Δ::KanMX slaΔ::HIS3* | pRS315-Neo1S221L | This study |
| BKJ845 | MATa his3Δ1 leu2Δ0 ura3Δ0 lys2Δ0neo1Δ::KanMX Sec7-3xGFP | pRS313-Neo1 | This study |
| BKJ846 | MATa his3Δ1 leu2Δ0 ura3Δ0 lys2Δ0neo1Δ::KanMX Sec7-3xGFP | pRS313-Neo1 S221L | This study |
| BKJ847 | MATa his3Δ1 leu2Δ0 ura3Δ0 lys2Δ0neo1Δ::KanMX Sec7-3xGFP | pRS313-Neo1 R247L | This study |
| BKJ848 | MATa his3Δ1 leu2Δ0 ura3Δ0 lys2Δ0neo1Δ::KanMX Sec7-3xGFP | pRS313-Neo1 S452Q | This study |
| BKJ849 | MATa his3Δ1 leu2Δ0 ura3Δ0 lys2Δ0neo1Δ::KanMX Sec7-3xGFP | pRS313-Neo1 P456A | This study |
| BKJ850 | MATa his3Δ1 leu2Δ0 ura3Δ0 lys2Δ0neo1Δ::KanMX iGFP-Tlg1 | pRS313-Neo1 | This study |
| BKJ851 | MATa his3Δ1 leu2Δ0 ura3Δ0 lys2Δ0neo1Δ::KanMX iGFP-Tlg1 | pRS313-S221L | This study |
| BKJ852 | MATa his3Δ1 leu2Δ0 ura3Δ0 lys2Δ0neo1Δ::KanMX iGFP-Tlg1 | pRS313-Neo1 R247L | This study |
| BKJ853 | MATa his3Δ1 leu2Δ0 ura3Δ0 lys2Δ0neo1Δ::KanMX iGFP-Tlg1 | pRS313-Neo1 S452Q | This study |

| | | | |
|---|---|---|---|
| BKJ854 | MATa his3Δ1 leu2Δ0 ura3Δ0 lys2Δ0neo1Δ::KanMX iGFP-Tlg1 | pRS313-Neo1 P456A | This study |

**Plasmids used in the study for Bacterial expression:**

| **Plasmid** | **Source** |
|---|---|
| pET28a-GFPSidC(614-743) | This Study |
| pET28a-GFPSidC(614-743)R652Q | This Study |
| His6-EGFP-PH-PLCD1 (Addgene Plasmid #183675) | (Walpole et al., 2022) |
| YIplac211-iGFP-TLG1 (Addgene Plasmid # 105261) | (Day et al., 2018) |
