# Supplementary material for: P4-ATPases control phosphoinositide membrane asymmetry and neomycin resistance
Source: Nat Cell Biol. 2025 Jul 11;27(7):1114–24. doi: 10.1038/s41556-025-01692-z (PMC12270916; doi:10.1038/s41556-025-01692-z)
Supplement: Supplementary file 2 — Reporting Summary [file 41556_2025_1692_MOESM2_ESM.pdf]

nature portfolio

Corresponding author(s): Todd R Graham

Last updated by author(s): Apr 21, 2025

Reporting Summary

Nature Portfolio wishes to improve the reproducibility of the work that we publish. This form provides structure for consistency and transparency in reporting. For further information on Nature Portfolio policies, see our Editorial Policies and the Editorial Policy Checklist.

Statistics

For all statistical analyses, confirm that the following items are present in the figure legend, table legend, main text, or Methods section.

n/a	Confirmed	
☐	☒	The exact sample size (*n*) for each experimental group/condition, given as a discrete number and unit of measurement
☐	☒	A statement on whether measurements were taken from distinct samples or whether the same sample was measured repeatedly
☐	☒	The statistical test(s) used AND whether they are one- or two-sided<br>*Only common tests should be described solely by name; describe more complex techniques in the Methods section.*
☐	☒	A description of all covariates tested
☒	☐	A description of any assumptions or corrections, such as tests of normality and adjustment for multiple comparisons
☐	☒	A full description of the statistical parameters including central tendency (e.g. means) or other basic estimates (e.g. regression coefficient) AND variation (e.g. standard deviation) or associated estimates of uncertainty (e.g. confidence intervals)
☐	☒	For null hypothesis testing, the test statistic (e.g. *F*, *t*, *r*) with confidence intervals, effect sizes, degrees of freedom and *P* value noted<br>*Give P values as exact values whenever suitable.*
☒	☐	For Bayesian analysis, information on the choice of priors and Markov chain Monte Carlo settings
☒	☐	For hierarchical and complex designs, identification of the appropriate level for tests and full reporting of outcomes
☒	☐	Estimates of effect sizes (e.g. Cohen's *d*, Pearson's *r*), indicating how they were calculated

Our web collection on statistics for biologists contains articles on many of the points above.

Software and code

Policy information about availability of computer code

Data collection	Cryo-EM data were collected using SerialEM v4.0. Fluorescence images were acquired using a Delta Vision Elite Imaging System (GE Healthcare Life Sciences, Pittsburgh, PA) 100X,1.4 NA oil immersion objective lens. The images were deconvoluted by softWoRx software (v7.0.0; GE Healthcare) or Images were acquired using Inverted LSM880-Airyscan (Zeiss) microscope equipped with a 63×,1.4 NA oil immersion objective lens followed by deconvolution using Zen Desk software.
Data analysis	cryoSPARC v4.2.1, Coot v0.9.8.3, ISOLDE v1.6, Phenix v1.20.1, MolProbity v4.5.1, UCSF ChimeraX v1.7. Images were analyzed and fluorescence intensity was quantified using ImageJ1.54g. GraphPad Prism 9.5.0.730

For manuscripts utilizing custom algorithms or software that are central to the research but not yet described in published literature, software must be made available to editors and reviewers. We strongly encourage code deposition in a community repository (e.g. GitHub). See the Nature Portfolio guidelines for submitting code & software for further information.

Data

Policy information about availability of data

All manuscripts must include a data availability statement. This statement should provide the following information, where applicable:

- Accession codes, unique identifiers, or web links for publicly available datasets
- A description of any restrictions on data availability
- For clinical datasets or third party data, please ensure that the statement adheres to our policy

The cryo-EM 3D map of the S. cerevisiae Neo1 in E2P state and bound with PI4P has been deposited in the Electron Microscopy Data Bank under accession code EMD-44850. The corresponding atomic model has been deposited in the Protein Data Bank under accession code 9BS1. Source data are provided with this paper. All other data supporting the findings of this study are available from the corresponding author on reasonable request.

Research involving human participants, their data, or biological material

Policy information about studies with human participants or human data. See also policy information about sex, gender (identity/presentation), and sexual orientation and race, ethnicity and racism.

Reporting on sex and gender	N/A
Reporting on race, ethnicity, or other socially relevant groupings	N/A
Population characteristics	N/A
Recruitment	N/A
Ethics oversight	N/A

Note that full information on the approval of the study protocol must also be provided in the manuscript.

Field-specific reporting

Please select the one below that is the best fit for your research. If you are not sure, read the appropriate sections before making your selection.

☒ Life sciences ☐ Behavioural & social sciences ☐ Ecological, evolutionary & environmental sciences

For a reference copy of the document with all sections, see nature.com/documents/nr-reporting-summary-flat.pdf

Life sciences study design

All studies must disclose on these points even when the disclosure is negative.

Sample size	The sample size for cryo-EM studies were determined by properties and qualities of the particles and also the number of particles available in each micrograph. For the current cryo-EM dataset, 6,166 raw micrographs were collected.<br>For cellular studies, No statistical methods was used to determined the sample sizes but our sample sizes are similar to those reported in the previous studies in the field. For all the cellular studies 3 independent biological isolates were used for the experiments. All the yeast growth assay, imaging were performed using 3 independent biological isolates. 3 Independent experiments were performed when siRNA studies were performed in the cell lines.<br>For fluorescence microscopy, No statistical methods was used to determined the sample sizes. Images from 20 cells were collected for 3 independent biological isolates or experiments.
Data exclusions	"Bad" raw particle images that did not produce 2D class averages or 3D class maps with defined features were excluded after 2D and 3D classifications. The criterion is empirical but is a standard image processing practice in the cryo-EM community.
Replication	Reproducibility resides in the large number of particles used to derive at the final 3D maps or 2D averages. The reliability and the resolution are measured by the Gold-standard Fourier shell correlation. Replication efforts with multiple refinement runs successfully yielded similar 3D maps. All the experiments were performed in the triplicates or 3 independent biological isolates were used.
Randomization	The allocation or selection of “good” and “bad” particles are determined by the computer program CryoSPARC based on the 2D templates or 3D volumes provided prior to running the program.
Blinding	The investigators cannot be blinded to the specific data points during data collection and analysis, because visual inspection is necessary to ascertain the data quality. There is no need for blinding in this type of study.

Reporting for specific materials, systems and methods

We require information from authors about some types of materials, experimental systems and methods used in many studies. Here, indicate whether each material, system or method listed is relevant to your study. If you are not sure if a list item applies to your research, read the appropriate section before selecting a response.

Materials & experimental systems

n/a	Involved in the study
☐	☒ Antibodies
☐	☒ Eukaryotic cell lines
☒	☐ Palaeontology and archaeology
☒	☐ Animals and other organisms
☒	☐ Clinical data
☒	☐ Dual use research of concern
☒	☐ Plants

Methods

n/a	Involved in the study
☒	☐ ChIP-seq
☒	☐ Flow cytometry
☒	☐ MRI-based neuroimaging

Antibodies

Antibodies used

Rabbit Anti-ATP9A antibody (ab234873, 1:1000)
Mouse Anti-β-Actin antibody (CST#3700, 1:10000)
Anti-Mouse lgG (H+L), HRP conjugate (W4021, 1:10,000 dilution) Lot: 0000459067 Promega (Madison, WI).
Anti-Rabbit lgG (H+L), HRP conjugate (W4011, 1:10,000 dilution) Lot: 0000529943 Promega (Madison, WI).

Validation

Rabbit Anti-ATP9A antibody (ab234873, 1:1000) Used in (PMID-36715683) https://www.abcam.com/products/primary-antibodies/atp9a-antibody-ab234873.html
Mouse Anti-β-Actin antibody (CST#3700, 1:10000) https://www.cellsignal.com/products/primary-antibodies/b-actin-8h10d10-mouse-mab/3700

Anti-Mouse lgG (H+L), HRP conjugate (W4021, 1:10,000) Lot: 0000459067 https://www.promega.com/products/protein-detection/primary-and-secondary-antibodies/anti_mouse-igg-h-and-l-hrp-conjugate/? catNum=W4021
Anti-Rabbit lgG (H+L), HRP conjugate (W4011, 1:10,000) Lot: 0000529943 https ://www. pro mega. com/products/protein-detection/primary-an d-seco n da ry-a nti bodies/anti-rabbit -igg-h-a nd-1-h rp-co n jugate/? catNum=W4011

Eukaryotic cell lines

Policy information about cell lines and Sex and Gender in Research

Cell line source(s)	HeLa cells (RRID:CVCL_0030), HEK293 cells (ATCC, RRID:CVCL_1573) .
Authentication	Cell lines obtained were not authenticated.
Mycoplasma contamination	Cell lines were not tested for mycoplasma contamination.
Commonly misidentified lines (See ICLAC register)	No misidentified cell lines were used in the study

Plants

Seed stocks

Report on the source of all seed stocks or other plant material used. If applicable, state the seed stock centre and catalogue number. If plant specimens were collected from the field, describe the collection location, date and sampling procedures.

Novel plant genotypes

Describe the methods by which all novel plant genotypes were produced. This includes those generated by transgenic approaches, gene editing, chemical/radiation-based mutagenesis and hybridization. For transgenic lines, describe the transformation method, the number of independent lines analyzed and the generation upon which experiments were performed. For gene-edited lines, describe the editor used, the endogenous sequence targeted for editing, the targeting guide RNA sequence (if applicable) and how the editor was applied.

Authentication

Describe any authentication procedures for each seed stock used or novel genotype generated. Describe any experiments used to assess the effect of a mutation and, where applicable, how potential secondary effects (e.g. second site T-DNA insertions, mosiacism, off-target gene editing) were examined.
