# Supplementary figures and images for: P4-ATPases control phosphoinositide membrane asymmetry and neomycin resistance
Source: Nat Cell Biol. 2025 Jul 11;27(7):1114–24. doi: 10.1038/s41556-025-01692-z (PMC12270916; doi:10.1038/s41556-025-01692-z)

Fig 3a: HeLa cell

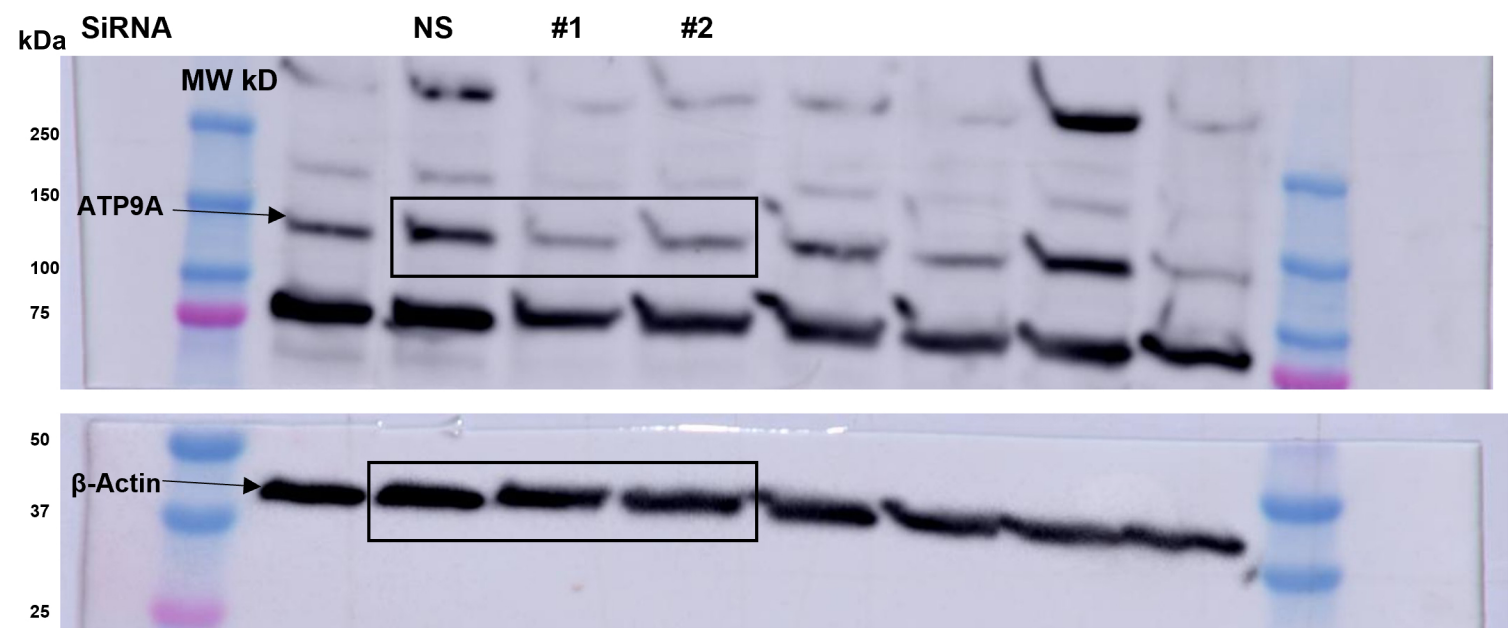

Fig 3c: HEK293 cell

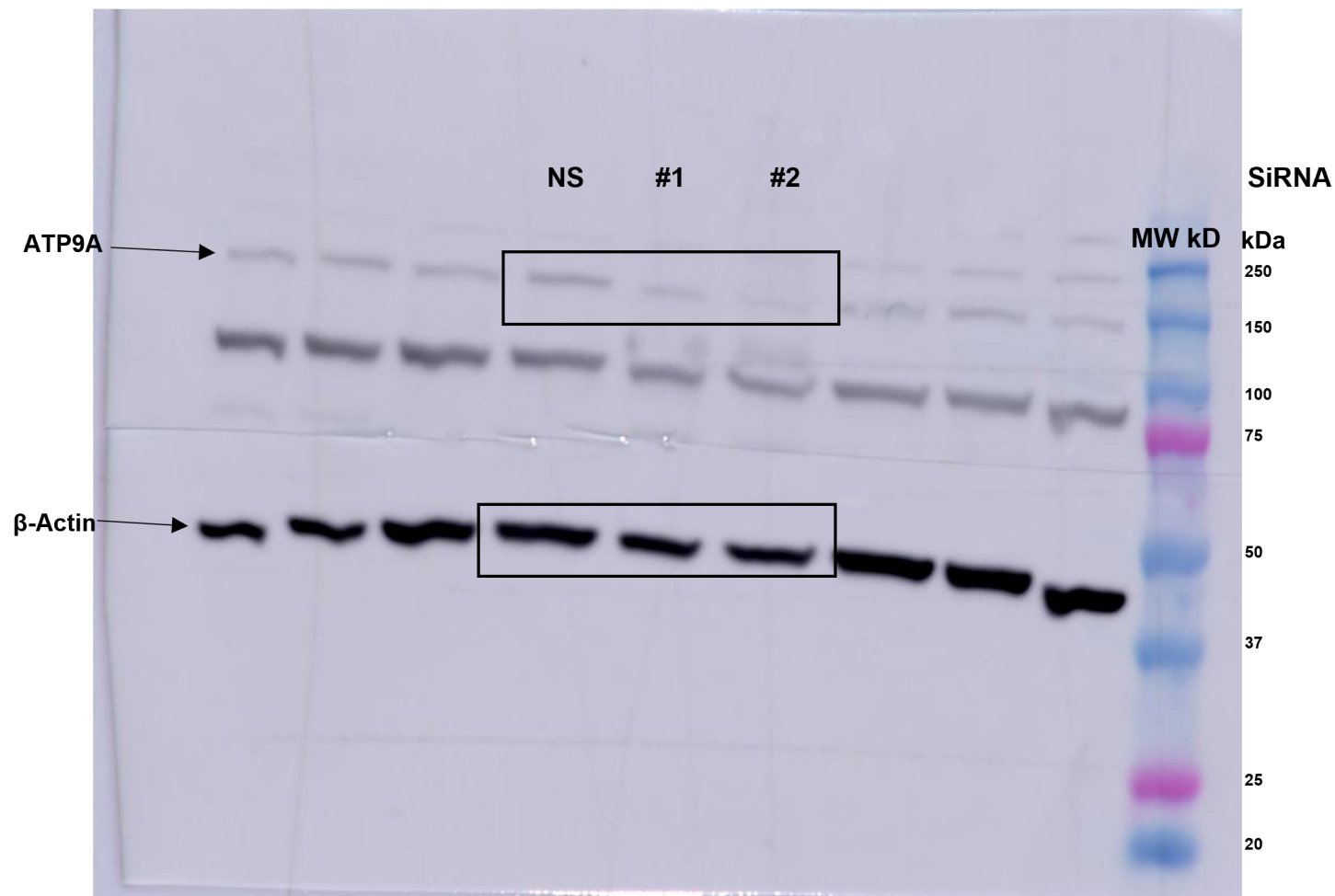

Supplement: Supplementary file 7 — Statistical source data, unprocessed western blots. [file 41556_2025_1692_MOESM7_ESM.pdf]

### Extended Data Fig 4b: Expression of ATP9A in HeLa cells and HEK293 cells

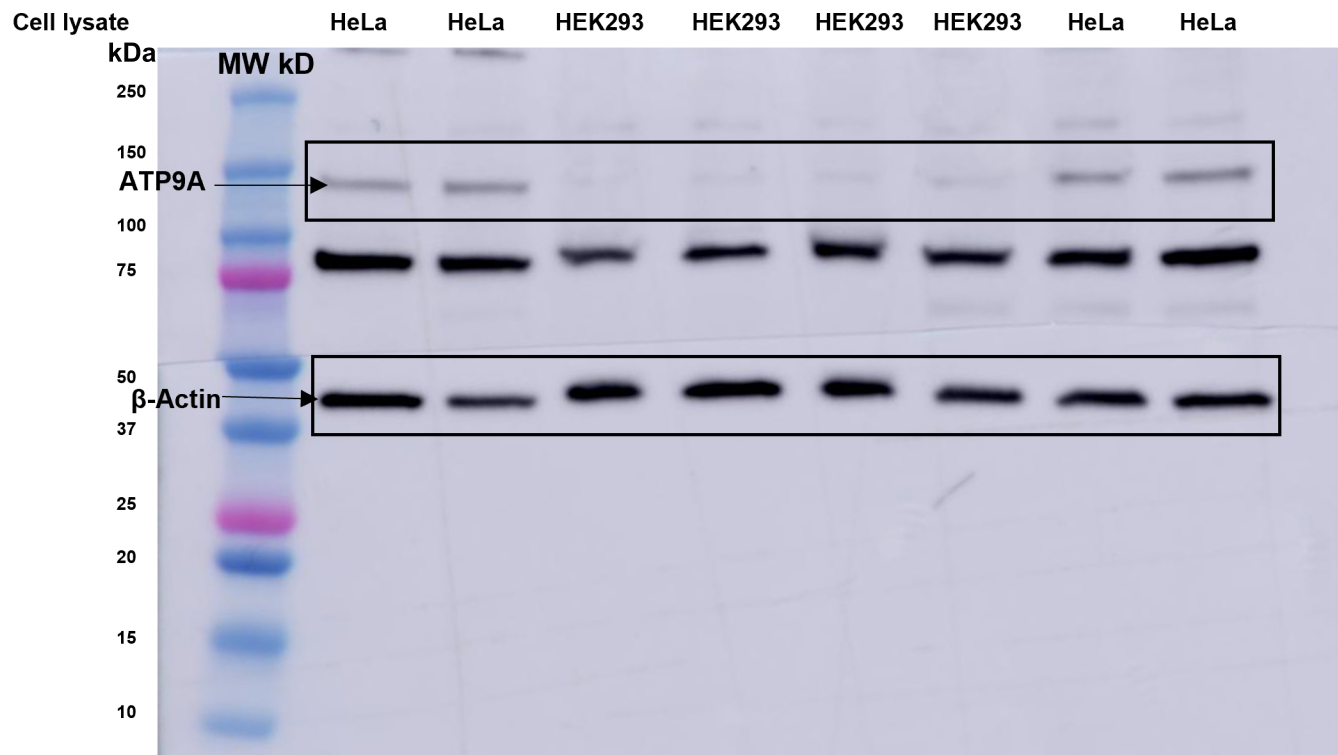

Supplement: Supplementary file 13 — Unprocessed western blots. [file 41556_2025_1692_MOESM13_ESM.pdf]
